# New genes involved in Angelman syndrome-like: Expanding the genetic spectrum

**DOI:** 10.1371/journal.pone.0258766

**Published:** 2021-10-15

**Authors:** Cinthia Aguilera, Elisabeth Gabau, Ariadna Ramirez-Mallafré, Carme Brun-Gasca, Jana Dominguez-Carral, Veronica Delgadillo, Steve Laurie, Sophia Derdak, Natàlia Padilla, Xavier de la Cruz, Núria Capdevila, Nino Spataro, Neus Baena, Miriam Guitart, Anna Ruiz

**Affiliations:** 1 Genetics Laboratory, UDIAT-Centre Diagnòstic, Parc Taulí Hospital Universitari, Institut d’Investigació i Innovació Parc Taulí I3PT, Universitat Autònoma de Barcelona, Sabadell, Spain; 2 Paediatric Unit, Parc Taulí Hospital Universitari, Institut d’Investigació i Innovació Parc Taulí I3PT, Universitat Autònoma de Barcelona, Sabadell, Spain; 3 Department of Clinical Psychology and Health Psychology, Universitat Autònoma de Barcelona, Bellatera, Barcelona, Spain; 4 CNAG‐CRG, Centre for Genomic Regulation (CRG), The Barcelona Institute of Science and Technology, Barcelona, Spain; 5 Neurosciences Area, Vall d’Hebron Institute of Research (VHIR), Universitat Autònoma de Barcelona, Barcelona, Spain; 6 Institució Catalana de Recerca i Estudis Avançats (ICREA), Barcelona, Spain; Odense University Hospital, DENMARK

## Abstract

Angelman syndrome (AS) is a neurogenetic disorder characterized by severe developmental delay with absence of speech, happy disposition, frequent laughter, hyperactivity, stereotypies, ataxia and seizures with specific EEG abnormalities. There is a 10–15% of patients with an AS phenotype whose genetic cause remains unknown (Angelman-like syndrome, AS-like). Whole-exome sequencing (WES) was performed on a cohort of 14 patients with clinical features of AS and no molecular diagnosis. As a result, we identified 10 *de novo* and 1 X-linked pathogenic/likely pathogenic variants in 10 neurodevelopmental genes (*SYNGAP1*, *VAMP2*, *TBL1XR1*, *ASXL3*, *SATB2*, *SMARCE1*, *SPTAN1*, *KCNQ3*, *SLC6A1* and *LAS1L*) and one deleterious *de novo* variant in a candidate gene (*HSF2*). Our results highlight the wide genetic heterogeneity in AS-like patients and expands the differential diagnosis.

## Introduction

Angelman syndrome (AS, OMIM #105830) is a neurogenetic disorder with a prevalence of about 1/15000 births. AS is characterized by severe developmental delay/intellectual disability (DD/ID) with absence of speech and distinctive dysmorphic craniofacial features such as microcephaly and wide mouth. Neurological problems include ataxia and seizures with specific electroencephalogram (EEG) abnormalities. The behavioral phenotype is characterized by happy disposition, frequent laughter, hyperactivity and stereotypies [1]. The consensus criteria for the clinical diagnosis of AS was proposed in 2005 by Williams et al., [1] which included a list of (i) consistent, (ii) frequent and (iii) associated features. However, clinical manifestations of AS can overlap with other diseases.

AS is caused by the loss of function in neuronal cells of the ubiquitin protein ligase E6-AP (E6-Associated Protein) encoded by the *UBE3A* gene, which is located on chromosome 15q11-q13 imprinted region. Methylation study of this region identifies 75–80% of AS patients including maternal deletion, paternal uniparental disomy (UPD) and imprinting center defects. Pathogenic or likely pathogenic variants in the *UBE3A* gene identify a further 10% of cases. However, for approximately 10–15% of clinically diagnosed AS patients, the genetic cause remains unknown (AS-like) [2].

Some of these AS-like patients present alternative clinical and molecular diagnoses in syndromes that have overlapping clinical phenotypes and that should be considered in the differential diagnosis of AS. AS differential diagnosis include single gene disorders such as Christianson syndrome (*SLC9A6)*, Rett syndrome (*MECP2*), Pitt Hopkins syndrome (*TCF4*), Kleefstra syndrome (*EHMT1*), Mowat-Wilson syndrome (*ZEB2*) or HERC2 deficiency syndrome (*HERC2*). Individuals affected by the above mentioned syndromes present severe DD, seizures, postnatal microcephaly, absent or minimal speech and sleep disturbances as AS patients [3–5].

In order to further identify the molecular defects in AS-like patients, whole exome sequencing (WES) was performed in a cohort of 13 parent-patient trios and one single patient with clinical features of AS and no molecular diagnosis. Pathogenic/likely pathogenic variants in known neurodevelopmental genes were found in 78,5% of patients while a deleterious variant in a new candidate gene was identified in another patient. Overall, our results show that 10–15% of patients with a clinical but with no molecular diagnosis of AS present alternative genetic alterations in genes not previously associated with AS, expanding the genetic spectrum of AS-like.

## Material and methods

### Patient samples

14 patients (7 girls and 7 boys) referred to the Angelman syndrome Unit at the Parc Taulí Hospital Universitari (Sabadell, Spain) were enrolled in the study. Patient 1 has also been included in another study [6]. The corresponding written informed consent was obtained from all parents of each participant. The study was approved by the institutional Ethics Committee of Institut d’Investigació i Innovació Parc Taulí I3PT (CEIC 2016/668).

The clinical diagnosis was made between ages 11 months and 8 years. All patients presented neurodevelopmental phenotypes suggestive of AS including severe global developmental delay, speech impairment and a behavioral phenotype that included apparent happy disposition as the most remarkable feature. AS negative testing included the analysis of the methylation status of the *SNURF-SNRPN* locus and Sanger sequencing and intragenic deletions/duplications analysis of the *UBE3A* gene. In addition, no alterations were detected by array comparative genomic hybridization (aCGH, ISCA 60 Kb, Agilent Technologies) and fragile X syndrome testing.

All the cases were sporadic and no other relevant findings were present in their family history.

### Whole-exome sequencing and variant interpretation

Trio WES of 13 patients and their parents was performed using the SureSelect Human All Exon V5+UTR kit (Agilent technologies). In patient 4, WES was performed only in the patient sample. Sequencing was performed on an Illumina Hiseq2000 platform (Illumina, San Diego, CA, USA) producing 2x100nt paired end reads at the National Centre of Genomic Analysis (CNAG-CRG, Barcelona, Spain). Raw data quality was assessed using FastQC software (v0.11.8, available at https://www.bioinformatics.babraham.ac.uk/projects/fastqc/) and an in depth analysis of each single generated FastQ file was performed to discard sequencing systematic errors and biases. On average, approximately 102.4 million reads per sample were generated during the sequencing process with an average GC content of 47.4% (standard deviation = 0.6%). Each sequenced base had on average a coverage of 67x and for each individual 87% of bases had a coverage >15. Raw reads were mapped to the human reference genome (hg19) using the Burrows-Wheeler aligner (BWA, v0.7.17-r1188) [7] and subsequently processed using the Genome Analysis Toolkit (GATK) pipeline in order to remove PCR duplicates and perform base quality score recalibration. Reads with RMS Mapping Quality (MQ) = 255, with bad mates or a Phred mapping quality <20 were filtered out, only bases with Phred quality score>18 were considered for variant calling and only variants with Phred-scaled confidence>10 were called. Variant discovery was performed using the Haplotype Caller tool and following the best practices for exome sequencing variant discovery of GATK (v4.0.11.0) [8]. On average, 21,170 exonic variants were detected for each individual, among which on average 9,570 were missense variants, 351 were loss of functions, 237 were non-frameshift variants and 10,606 were synonymous variants. The remaining exonic variants were classified as “unknown” by ANNOVAR.

All exome variants were first checked against a *de novo* followed by an X-linked and autosomal recessive model of inheritance. In order to detect *de novo* variants, only variants with valid genotype and genotype quality ≥20 in all the trio members were considered. Variants having a read depth lower than 5 in the parents or lower than 10 in the patients were discarded. Only variants that were heterozygous in the patients but homozygous for the reference allele in the parents were considered. Finally, putative *de novo* variants were filtered considering only those showing the alternative allele in more than 10% of the reads.

According to an autosomal recessive model of inheritance, annotated variants were filtered for allele frequencies<0.02 in the gnomAD database (v2.1.1) and their predicted impact on the protein. X-linked variants were filtered for allele frequencies <0.001 and their predicted impact on the protein.

Both *de novo* and recessive variants were annotated using ANNOVAR (v:16.04.2018) [9] a tool suited for functional annotation of variants detected from high-throughput sequencing data and assessing the impact of missense variants leveraging several *in silico* tools (S1 Table). Splice site variants were evaluated using the software Human Splicing Finder [10].

Sanger sequencing of the candidate variants was performed in the patients and the parents in order to confirm the presence of the variant and the pattern of inheritance. Variants were classified following the American College of Medical Genetics and Genomics and the Association for Molecular Pathology (ACMG/AMP) guidelines [11]. Pathogenic and likely pathogenic variants have been submitted to ClinVar [12].

### Real time quantitative PCR (RTqPCR) analysis

RNA was extracted using the Biostic Blood Total RNA Isolation Kit sample (MO BIO laboratories, Inc) and cDNA was obtained using the PrimeScript^TM^RT reagent Kit (Takara). RTqPCR gene expression analysis was performed in triplicate using the Taqman probes HSF2-Hs00988309_g1 and GADPH-Hs02758991_g1 for normalization (Applied Biosystems).

## Results

Identified variants were first filtered according to a dominant *de novo* model of inheritance. Variants in genes known to be involved in neurodevelopmental diseases were prioritized and confirmed to be *de novo*. Overall, 10 *de novo* (*SYNGAP1*, *VAMP2*, *TBL1XR1*, *ASXL3*, *SATB2*, *SMARCE1*, *SPTAN1*, *KCNQ3* and *SLC6A1)* and 1 X-linked (*LAS1L*) pathogenic and likely pathogenic variants were confirmed in 11 patients, leading to a diagnostic yield of 78,5% (Table 1). These variants were located in 10 different genes previously reported to be associated with neurodevelopmental disorders [6,13–21].

**Table 1:** Pathogenic and likely pathogenic variants identified in AS-like patients.

Patient	Gene	NM number	Nucleotide change	Amino acid change	Variant Type	Pattern of inheritance	ACMG/AMP Classification	Described before	Protein function
1	*VAMP2*	NM_014232.2	c.128_130delTGG	p.Val43del	In-frame	*De novo*	Pathogenic	Yes Salpietro et al., 2019 [6]	*VAMP2* is a member of the SNARE family of proteins, which are involved in membrane fusion of synaptic vesicles.
2	*SYNGAP1*	NM_006772.2	c.1861C>T	p.Arg621*	Nonsense	*De novo*	Pathogenic	No	*SYNGAP1* is a RAS-GTPase-activating protein with a critical role in synaptic development, structure, function and plasticity.
3	*TBL1XR1*	NM_024665.5	c.1000T>C	p.Cys334Arg	Missense	*De novo*	Likely pathogenic	No	*TBL1XR1* is part of the repressive NCoR/SMRT complex acting as a transcriptional regulator.
4	*TBL1XR1*	NM_024665.5	c.1043A>G	p.His348Arg	Missense	*De novo*	Likely pathogenic	No	
5	*SATB2*	NM_001172509.1	c.1826delA	p.Asp609Alafs*15	Frameshift	*De novo*	Pathogenic	No	*SATB2* participates in chromatin remodeling and transcription regulation.
6	*KCNQ3*	NM_004519.3	c.688C>T	p.Arg230Cys	Missense	*De novo*	Pathogenic	Yes Decipher and Miceli F et al., 2015, Sands TT et al., 2019 [20,22,23]	*KCNQ3* is a voltage-gated potassium channel subunits that underlay the neuronal M-Current.
7	*SMARCE1*	NG_032163.1 (NM_003079.4)	c.237+1G>T	p.Ala53_Lys79del	Splice site	*De novo*	Likely pathogenic	Yes Aguilera et al., 2019 [24]	*SMARCE1* is part of the SWI/SNF chromatin remodeling complex involved in transcriptional activation.
8	*SPTAN1*	NM_001130438.2	c.6592_6597dupCTGCAG	p.Leu2198_Gln2199dup	In-frame	*De novo*	Likely pathogenic	No	*SPTAN1* is an α-ΙΙ spectrin involved in stabilization and activation of membrane channels, transporters and receptors.
9	*ASXL3*	NM_030632.2	c.3106C>T	p.Arg1036*	Nonsense	*De novo*	Pathogenic	Yes Kuechler A et al., 2017 [25]	*ASXL3* plays a role in the regulation of gene transcription and histone deubiquitination.
10	*LAS1L*	NM_031206.4	c.1237G>A	p.Gly413Arg	Missense	X-linked	Likely pathogenic	No	*LAS1L* is involved in the 60S ribosomal subunit synthesis and maturation of 28S rRNA.
14	*SLC6A1*	NM_003042.3	c.889G>A	p.Gly297Arg	Missense	*De novo*	Pathogenic	Yes Carvill GL et al., 2015 [26]	*SLC6A1* gene encodes for the GAT-1 GABA transporter.

**Fig 1:**
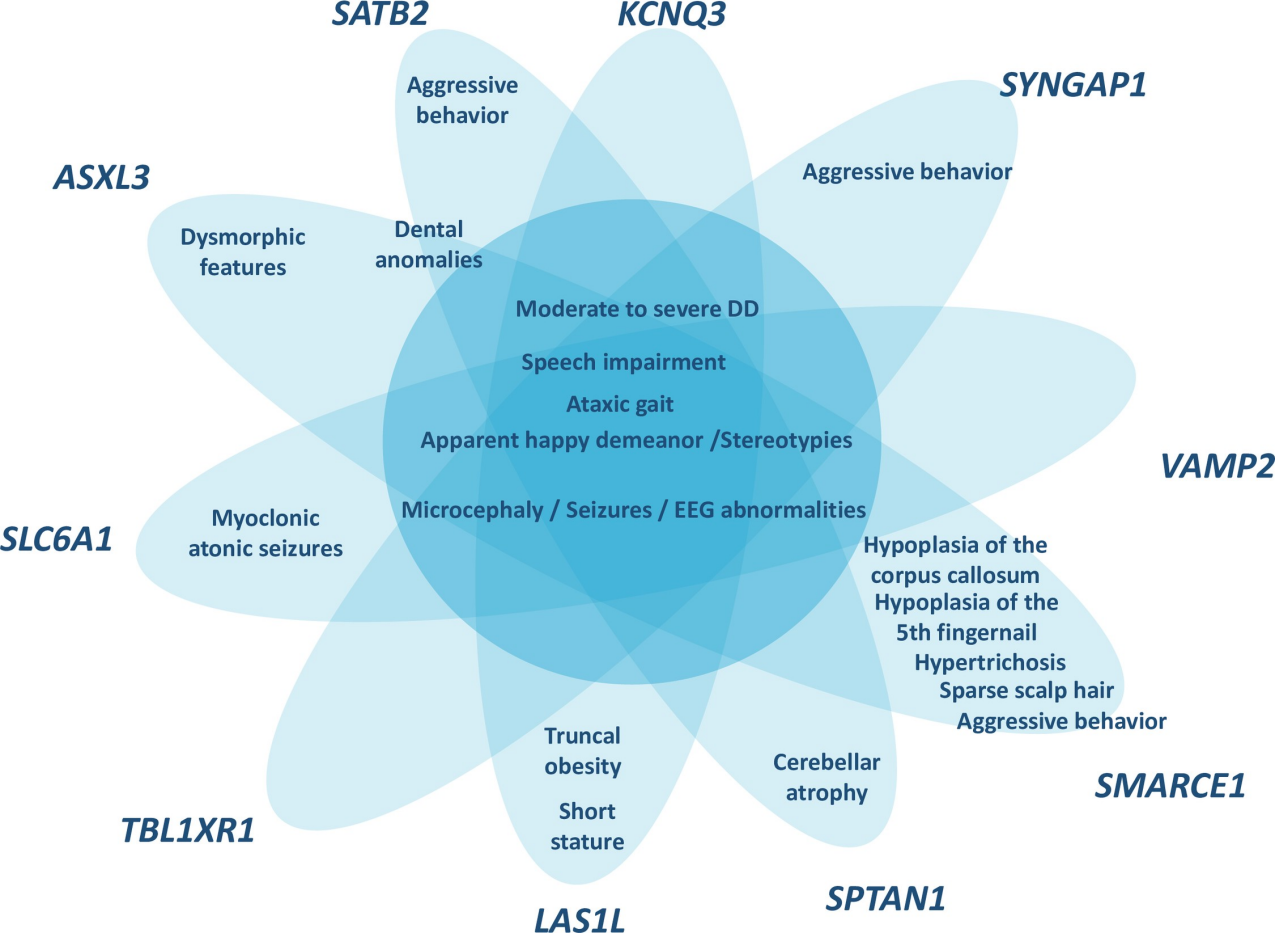
Schematic representation of the phenotypic overlap between the patients with pathogenic/likely pathogenic variants genes and the AS phenotype. In the middle of the figure the core AS features present in all the patients while in the tips the clinical features present in the patients that are associated with the gene identified.

Clinical re-evaluation of patients at the time of the molecular diagnosis (ages between 9–38 years) showed that all patients met the consistent clinical features of AS (Table 2), except for ataxia of gait which was present in 9 of 14 patients. Even though the ataxia of gait is considered a consistent feature in AS patients, a recent review shows that it ranges from 72,7% to 100% according to the genetic etiology [27]. Additional clinical features identified in the clinical re-evaluation of patients were analyzed taking into account the clinical phenotype described for the genes identified. The presence of specific clinical features associated with the new genes were confirmed for some of the patients. In short, cerebellar atrophy for *SPTAN1* [13], hypoplasia of the corpus callosum, hypoplasia of the 5^th^ finger nail, hypertrichosis, sparse scalp hair and aggressive behavior for *SMARCE1* [21], truncal obesity and short stature for *LAS1L* [16], myoclonic atonic seizures for *SLC6A1* [15], aggressive behavior for *SYNGAP1* [17], dysmorphic features and dental anomalies for *ASXL*3 [14] and aggressive behavior and dental anomalies for *SATB2* [19] (Fig 1).

**Table 2:** Characteristics of AS-like patients at clinical re-evaluation.

Patient	Gender	Age at molecular diagnosis (years)	Consistent features present in 100% of patients with AS				Frequent features present in more than 80% of AS affected individuals			Associated features present in 20–80% of AS affected individuals	Additional clinical features
			Severe developmental delay	Speech impairment	Ataxia or unsteady gait	Apparent happy demeanor/Stereotypies	Microcephaly	Seizures	Abnormal EEG		
1	M	14	+	+ (5–10 words)	+	+/+	-	+	+	Sleep disorder, hypotonia	Congenital torticolis, bruxism, aggressive behavior
2	F	19	+	+ (less than 5 words)	-	+/+	-	+	+	Sleep disorder, feeding problems, kyphoscoliosis	Aggressive behavior
3	F	12	+	+ (Absent speech)	+	-/+	-	+	+	Hypotonia	Aggressive behavior
4	F	9	+	+ (Absent speech)	+	+/+	+ (Relative)	-	+	Hypotonia, feeding problems	-
5	F	20	+	+ (5–10 words)	+	+/-	-	-	+	-	Dental anomalies, auto and hetero-aggressive behavior
6	F	18	+	+ (Absent speech)	-	+/-	+ (Relative)	+	-	Scoliosis	-
7	M	15	+	+ (less than 5 words)	+	+/+	-	+	+	Feeding problems, wide mouth, hypotonia	Sparse scalp hair, hypertricosis in the back and hypoplasia of the corpus callosum, hypoplasic 5^th^ fingernail, auto and hetero-aggressive behavior
8	F	14	+	+ (More than 20 words)	+	+/-	+	-	-	Hypotonia	Cerebellar atrophy
9	M	38	+	+ (Absent speech)	+	+/+	+ (Relative)	+	+	Hypotonia, feeding problems (esophageal reflux), sleep disorder	Dental anomalies, bruxism, episodic hyperventilation
10	M	7	+	+ (less than 5 words)	-	+/-	+ (Relative)	-	-	Wide spaced teeth, brachycephaly	Truncal obesity, short stature
11	F	14	+	+ (less than 5 words)	-	+/+	+ (Relative)	+	-	Feeding problems (dysphagia)	-
12	M	24	+	+ (5–10 words)	-	+/+	+	+	NA	Strabismus, sleep disorder, kyphoscoliosis	Hypoplasia of the corpus callosum, abnormal behavior, hypothyroidism, bruxism
13	M	9	+	+ (Absent speech)	+	+/+	+ (Relative)	+	-	Sleep disorder, hypotonia	Episodic hyperventilation, mild subcortical atrophy
14	M	13	+	+ (Absent speech)	+	+/+	-	+	+	Sleep disorder, wide-spaced teeth	Myoclonic atonic seizures, bruxism

M, Male; F, Female; +, present; -, not present; NA, non-available data.

However, not all patients presented all the clinical features associated with the genes identified. Unsteady gait and hypotonia were not present in patient carrying the pathogenic variant in *SYNGAP1* [17]. The patient harboring a pathogenic variant in *SATB2* did not show sialorrhea and feeding difficulties [19]. Finally, the ataxia of gait, stereotypies and hypotonia were not observed in the patient with a pathogenic variant in *KCNQ3* [20].

A novel candidate variant was identified in a gene not previously associated with neurodevelopmental disorders. The identified variant is a *de novo* frameshift deletion c.456_459delTGAG (NM_004506.3), p.(Ser152Argfs*40) in *HSF2* gene in patient 12. The variant has not been reported before and is not present in the gnomAD database (version 2.1.1).

UBE3A has been shown to have both nuclear and cellular functions mainly through its ubiquitin protein-ligase activity [28]. UBE3A interacts with most of the components of the proteasome [29] regulating the activity of signal transduction pathways such as Wnt signaling that regulates central nervous system development [30–32] and synaptic plasticity in both excitatory and inhibitory GABAergic axon terminals [33–36]. At the nucleus, UBE3A has been shown to regulate chromatin structure, DNA methylation and transcriptional regulation [37–40]. Interestingly, 8 out of the 10 genes found mutated in this study are mainly involved in synapsis (*VAMP2*, *SYNGAP1*, *SLC6A1* and *KCNQ3*) [41–44] and chromatin remodeling or transcription regulation (*TBL1XR1*, *SATB2*, *SMARCE1* and *ASXL3)* [45–48].

## Discussion

We identified causal variants in 11 out of 14 patients with an AS-like phenotype. The global yield diagnostic of WES in this is study is 78,5%, which is higher to what has been reported in the literature for other neurodevelopmental disorders (24–68%) [49]. The results of WES led to the identification of 10 new genes that cause an AS-like phenotype (*SYNGAP1*, *VAMP2*, *TBL1XR1*, *ASXL3*, *SATB2*, *SMARCE1*, *SPTAN1*, *KCNQ3*, *SLC6A1* and *LAS1L*), all of them previously associated with other neurodevelopmental disorders. In addition, we propose *HSF2* (Heat Shock Factor) as a new candidate gene for the AS-like phenotype. Although *HSF2* has not been previously associated with any human disease, the gene is highly expressed in the brain (Data source: GTEx Analysis Release V8, dbGaP Accession phs000424.v8.p2 [50]) and is highly intolerant to loss of function variation (pLI 0.92). Quantification of the mutated allele in mRNA showed a reduction in the allele carrying the frameshift variant (S1A Fig), suggesting the activation of the nonsense-mediated mRNA decay (NMD) machinery [51] and supporting a loss of function mechanism of disease for the *HSF2* gene. Expression analysis of *HSF2* in blood mRNA also showed a reduction in *HSF2* expression in the patient compared to control (p-value 0.014) (S1B Fig). However, other tissues should be examined to clearly demonstrate the activation of NMD and a loss of function mechanism for the *HSF2* variant.

Furthermore, *HSF2* knockout mice show defects in the development of the central nervous system and spermatogenesis [52,53]. The identification of additional patients with loss of function variants in *HSF2* and functional studies in neural cells will contribute to elucidate the role of *HSF2* in the AS-like phenotype.

*De novo* variants have been described to account for approximately half of the genetic architecture of severe developmental disorders [54]. In our cohort, 10 of the 11 pathogenic and likely pathogenic variants were *de novo*, accounting for 90% of diagnosis and highlighting the power of using trio-WES for the molecular diagnosis of severe developmental disorders. Only in one case, the X-linked variant in *LAS1L* was inherited from the mother, who was a healthy carrier (data not shown).

All patients had received a suspected clinical diagnosis of AS. In the majority of our patients (12/14) the initial diagnosis was done during infancy or early childhood (before five years old). At the time of initial diagnosis, all of them presented severe global DD and speech impairment in addition to the characteristic happy disposition. Clinical re-evaluation at the time of molecular diagnosis confirmed the clinical diagnosis of AS (Table 2). In addition, other clinical features manifested during growth were then associated with the new identified genes. Pathogenic/likely pathogenic variants in *VAMP2*, *KCNQ3*, *SMARCE1*, *SATB2*, *SYNGAP1*, *SLC6A1*, *ASXL3*, *SPTAN1*, *TBL1XR1* and *LAS1L* genes are associated with neurodevelopment disorders that overlap with AS and whose features have been defined in the last years [6,13–19,21]. Finally, not all patients presented all the clinical features associated with the genes identified. This clinical variability, possibly due to the different pathogenicity strength of the genetic variants, differences in genetic background and to non-genetic environmental factors, makes the clinical diagnosis challenging.

Lack of molecular diagnosis in 10–15% of clinically diagnosed AS patients has been used to define the AS-like group. Our trio based WES approach demonstrates that the majority of these patients (78,5%) are carriers of pathogenic variants in genes involved in neurodevelopmental disorders whose features overlap with AS (Fig 1), highlighting the wide genetic heterogeneity in AS-like patients and expanding the differential diagnosis. Likewise, other studies have recently described new genes associated with Angelman-like phenotypes such as *HIVEP2* [55] or *UNC80* [56].

*TBL1XR1*, *SATB2*, *SMARCE1* and *ASXL3* are known transcriptional regulators acting through chromatin modification [46,57,58]. UBE3A has been shown to be present in euchromatin-rich nuclear domains indicating that it may influence neuronal physiology by regulating chromatin and gene transcription [59]. RNA-seq studies of *UBE3A* loss in rat cortex, mice hippocampus and SH-SY5Y cells have shown differential gene expression of *KCNQ3* [60], *SMARCE1*, *HSF2* [38], *SPTAN1* and *SATB2* [39] suggesting that these genes may be transcriptionally regulated by UBE3A. Moreover, *UBE3A* gain and loss in human SH-SY5Y cells has been shown to have significant effects on DNA methylation and chromatin modification in genes involved in transcriptional regulation and brain development including *SATB2*, *ASXL3*, *SMARCE1* and *TBL1XR1* [38]. Overall, these evidences suggest common deregulated pathways between new identified Angelman-like genes and UBE3A. In addition, UBE3A has been shown to localize in axon terminals suggesting it locally regulates individual synapses. Five of the genes identified here, *VAMP2*, *SYNGAP1*, *SLC6A1* and *KCNQ3* are known to be involved in synapse function suggesting they may be regulated by UBE3A, as has already been demonstrated for *KCNQ3* [60].

Except for the *SYNGAP1* gene, none of the genes identified here have been previously described in the differential diagnosis of AS [61]. We propose the genes identified in this study should be included in the AS differential diagnosis and that trio WES should be considered as first line approach for the molecular diagnosis of AS-like patients. A high rate of diagnosis is essential since it contributes to more appropriate clinical patient surveillance as well as family genetic counseling.

## Supporting information

S1 Fig Quantification of *HSF2* mRNA transcripts suggest that variant c.456_459delTGAG is sensitive to nonsense-mediated mRNA decay (NMD). **A)** Sanger sequencing of a fragment encompassing variant c.456_459delTGAG from patient 12 and a control sample shows a reduction in the percentage of the allele with the variant in the cDNA compared to DNA. The sequence corresponds to the reverse strand. **B)** qPCR analysis of *HSF2* gene expression in patient 12 and a control sample normalized to *GAPDH* shows less *HSF2* expression in patient 12 (* p-value 0.014).
(TIF)

S1 Table Missense, in-frame and splice site variant predictors. Prob. DA, Probably Damaging; Pos. DA, Possibly Damaging; D, Deleterious; M, Medium; H, High; NA, Not Available.
(TIF)
