# Supplementary figures and images for: New genes involved in Angelman syndrome-like: Expanding the genetic spectrum
Source: PLoS One. 2021 Oct 15;16(10):e0258766. doi: 10.1371/journal.pone.0258766 (PMC8519432; doi:10.1371/journal.pone.0258766)

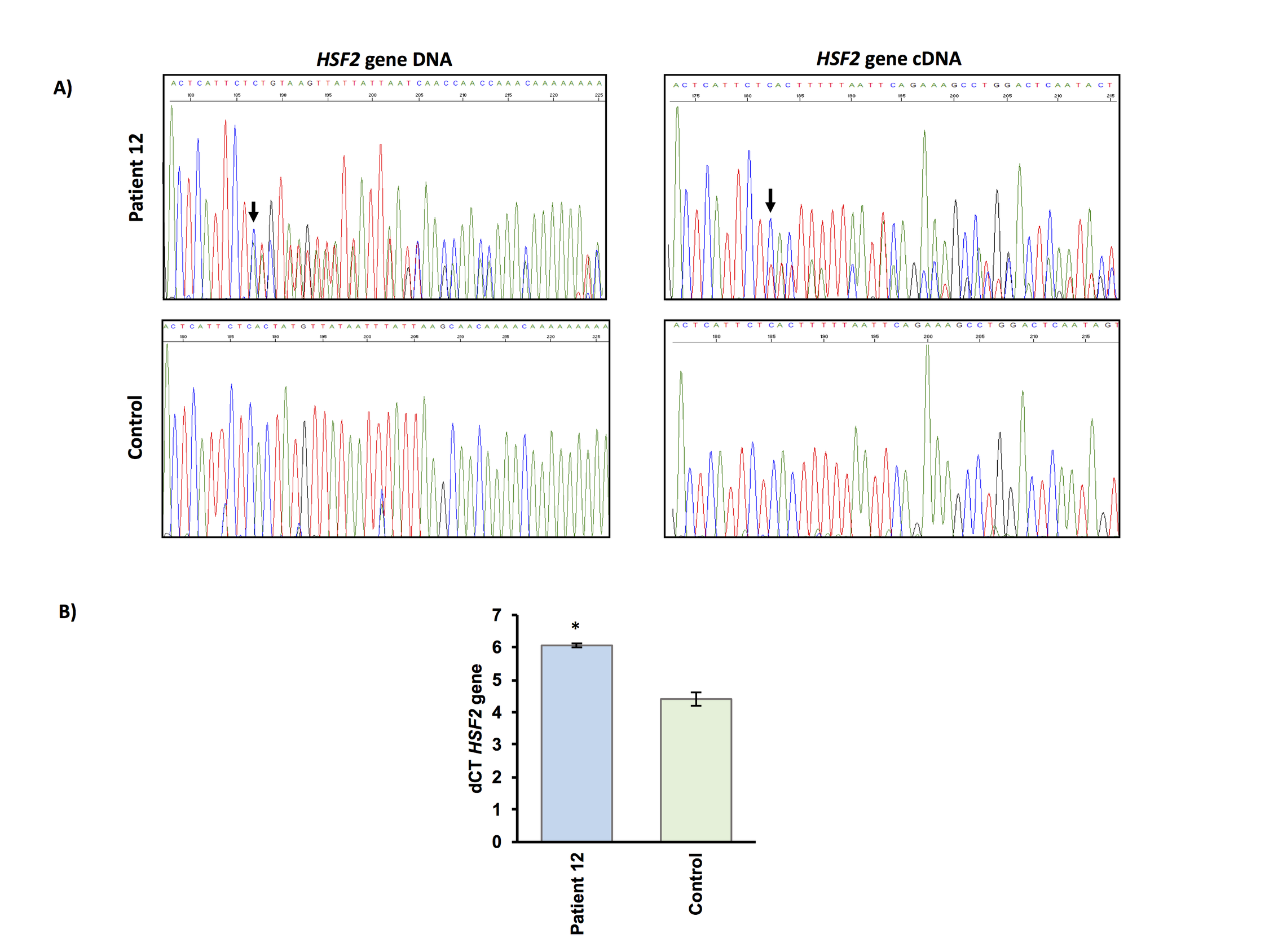

Supplement: S1 Fig — A) Sanger sequencing of a fragment encompassing variant c.456_459delTGAG from patient 12 and a control sample shows a reduction in the percentage of the allele with the variant in the cDNA compared to DNA. The sequence corresponds to the reverse strand. B) qPCR analysis of HSF2 gene expression in patient 12 and a control sample normalized to GAPDH shows less HSF2 expression in patient 12 (* p-value 0.014). (TIF) [file pone.0258766.s001.tif]

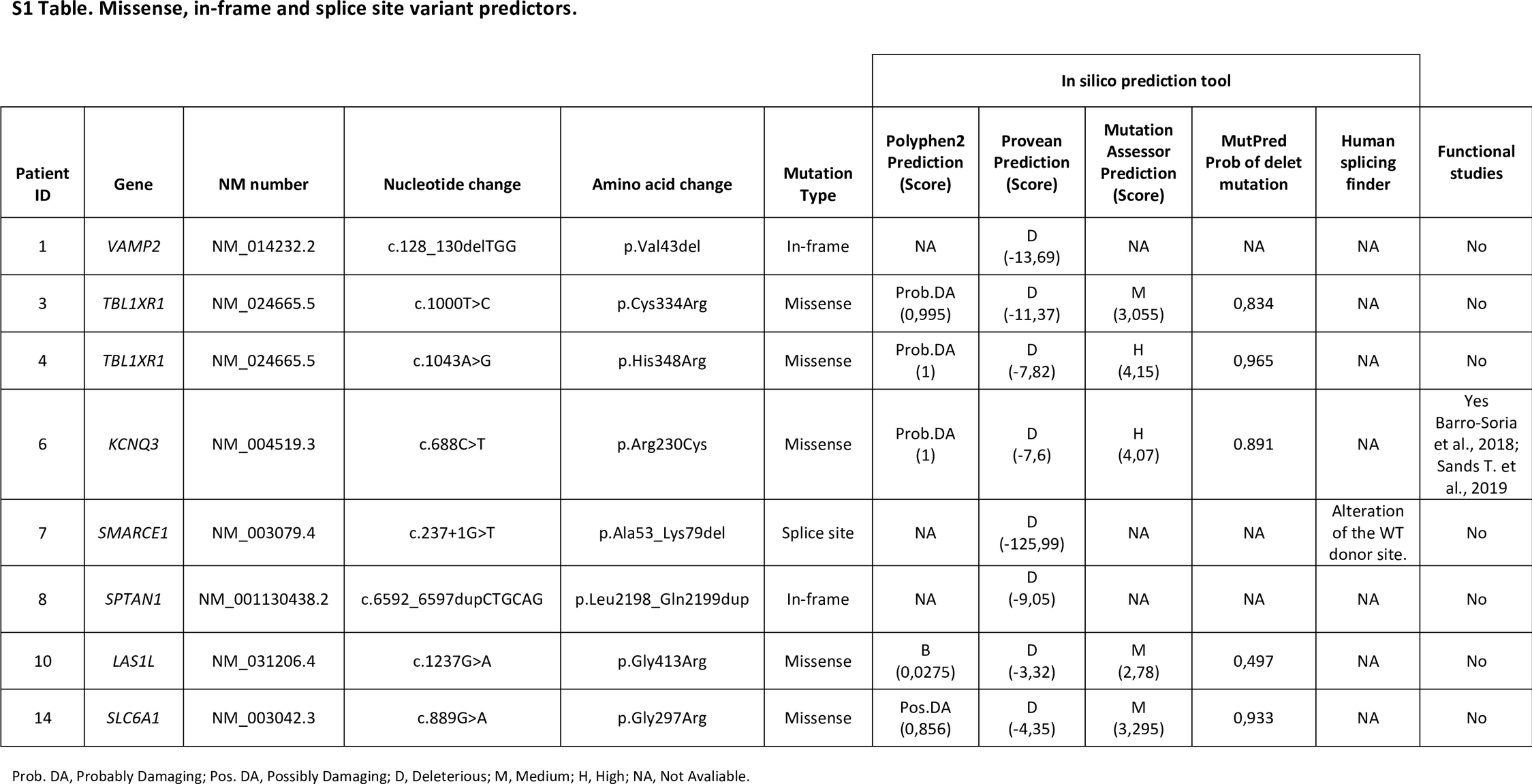

Supplement: S1 Table — Prob. DA, Probably Damaging; Pos. DA, Possibly Damaging; D, Deleterious; M, Medium; H, High; NA, Not Available. (TIF) [file pone.0258766.s002.tif]
